# Challenges with couples HIV counselling and testing among black MSM students: perspectives of university students in Durban, South Africa

**DOI:** 10.1080/17290376.2022.2101511

**Published:** 2022-07-30

**Authors:** Geogina Charity Gumindega, Pranitha Maharaj

**Affiliations:** School of Built Environment and Development Studies, University of KwaZulu-Natal, Durban, South Africa

**Keywords:** MSM, university students, risky behaviours, HIV prevention, couples testing

## Abstract

Research suggests that HIV infections among men who have sex with men (MSM) are acquired from primary partners, yet MSM continually fail to take part in couples HIV counselling and testing (CHCT). To identify factors that inhibit MSM in universities from regularly testing for HIV with their sexual partners, this study considered the perspectives and experiences of 15 MSM students in Durban, South Africa. The findings show that despite appreciating the value of couple testing it is relatively uncommon. MSM resist doing so with their casual partners as this would presumably signal an intention to advance the relationship beyond the short-term. Other barriers included; experienced and perceived homophobia at public testing centres, trust-based assumptions that primary partners need not test for HIV and fear of discord. They also employed alternative strategies to purportedly determine their casual and primary partners’ status in the absence of CHCT. Alternative strategies include; initiating sexual relationships with casual partners whose sexual history is known and making use of home-based testing kits to avoid CHCT at public testing centres. These findings emphasise the need for LGBTIQ-friendly couple-based approaches as a necessary component of HIV prevention interventions among MSM in universities.

## Introduction

HIV testing remains a key component of global prevention efforts (UNAIDS, 2016). This is particularly true among key populations such as men who have sex with men (MSM) who contribute 8% to the disproportionate number of new infections each year (UNAIDS, 2016). While it is important to ensure that prevention efforts centred around HIV testing and early diagnosis are strategically implemented to lower the prevalence of HIV rates in key populations, existing research has established that very few MSM in the developing world who are already infected know their HIV status let alone their partners’ HIV status (Beyrer et al., 2012; Sandfort, Knox, Collier, Lane, & Reddy, 2015). The World Health Organization (WHO) argues that couples HIV counselling and testing (CHCT) is an integral part of HIV testing procedures as it ensures mutual agreement in terms of HIV prevention and sexual health between individuals and their sexual partners (WHO, 2012). Some of the identified benefits of CHCT in relation to HIV prevention include the prevention of HIV transmission in couples as well as external partners through the use of condoms, early or increased uptake and adherence to antiretroviral therapy (ART), increased relationship cohesion which leads to normalisation of HIV positive status and decreased stigma and intimate partner violence (Farquhar et al., 2004; WHO, 2012).

The majority of studies on voluntary counselling and testing for couples in sub-Saharan Africa have focused on heterosexual men and women (Hailemariam, Nathan, Seifu, & Rawstorne, 2020; Maman et al., 2003; Matovu, 2017; Painter, 2001). Furthermore, much of the research has been done on married and cohabiting partners, which neglects CHCT among vulnerable populations such as MSM who are less likely to be living with their male partners due to the homophobic and heteronormative nature of sub-Saharan African communities (Hailemariam et al., 2020).

While extensive research has been done on HIV serodiscordance among men who have sex with men, some studies have shown that partnered MSM are not likely to test with their partners. Few identified studies have explored CHCT and its associated barriers among MSM and their partners (Remien, Carballo-Dieguez, & Wagner, 1995; Stephenson et al., 2011; Stephenson, Rentsch, & Sullivan, 2012; Sullivan et al., 2014). South African studies have established high levels of acceptability of CHCT among MSM (Stephenson et al., 2012). HIV testing rates remain lower among those who have a main partner than those who are single (Stephenson et al., 2012; Stephenson, Chard, Finneran, & Sullivan, 2014). The reasons for this include a perceived low risk among MSM who have a main partner as well as the effect of relationship dynamics on HIV testing behaviour (Mitchell & Horvath, 2013).

CHCT should be understood in various socio-economic contexts. This study approaches HIV testing in the context of MSM in South African universities. According to HEAIDS (2014), the average reported testing rates among MSM in institutions of higher learning are greater than the general population. No identified studies have investigated the HIV testing behaviours of MSM studying at universities and their partners. MSM couples in universities present unique challenges to prevention efforts yet CHCT is perceived to be not applicable to them because they are less likely to be cohabiting and engaging with a regular partner which leaves them open to multiple and casual sexual partnerships (HEAIDS, 2014). Universities create conducive environments for sexual experimentation and HIV-risky behaviours. This makes tertiary students an important group of interest as they belong to age groups that are experiencing freedom from parental guidance for the first time in their lives (Chanakira, O’Cathain, Goyder, & Freeman, 2014). Although, as highlighted earlier, MSM in universities are less likely to have regular partners, this study seeks to understand their past, present or future behaviours regarding their willingness to undergo CHCT.

This present study conceptualises the term MSM as a sexual behaviour rather than a sexual identity. As pointed out by Boellstorff (2011), some men have sexual intercourse with other men yet they do not identify as gay, homosexual or bisexual, thus the use of the term in this study does not point to any sexual identity or orientation but rather it is used to point to a behaviour. A report on MSM in South Africa (Scheibe et al., 2015, pp. xii), states that the term is used to ‘sex between people who were born biologically male. The term does not make reference to sexual identity nor sexual orientation as many MSM sometimes do not identify with a specific sexual orientation’.

The approach of the study to the MSM category as consisting of a range of sexual orientations may seemingly make the research design conceptually limited however, it is important to consider that care should be taken in defining MSM as a homogeneous group and confusing same-sex behaviour with sexual orientation. The definition of MSM used in this study distinguishes between behaviour and orientation as it is behaviour that puts one at risk. For this reason, this current study was conducted based on common behaviours i.e. risky sexual practices among men who have sex with other men regardless of their sexual orientation. It is also important to consider that student MSM are a minority population that encounter persistent discrimination and marginalisation in South African universities (Nduna, Mthombeni, Mavhandu-Mudzusi, & Mogotsi, 2017). Conceptual strictness in the use and definition of the term MSM as an independent sexual category rather than a behaviour would have limited this study.

This study aims to identify factors that inhibit MSM from regularly testing for HIV with their sexual partners. This study draws on interviews to better understand their perspectives and experiences of HIV testing with their sexual partners. The study described in this paper contributes to the growing body of literature advocating for CHCT which is contextually important with respect to high-risk population sub-groups such as MSM in universities.

### Study context

This study was conducted at the University of KwaZulu-Natal’s (UKZN) Durban campuses situated in the province of KwaZulu-Natal in South Africa. Despite being the second largest province, KwaZulu-Natal is also one of the poorest provinces in the country. According to a UNAIDS (2018) report, the province is an ‘HIV hotspot’ where 40.8% of adults are estimated to be living with HIV and people living in this province have a 46% higher risk of HIV infection than those living outside KwaZulu-Natal (UNAIDS, 2018). In the university context, the HIV prevalence rate in tertiary institutions has been found to be lower than the national average as this group consists of people with high levels of education and better access to information regarding HIV (HEAIDS, 2010). Durban has an unusually high HIV infection rate among adult MSM (Cloete et al., 2014; Rispel, Metcalf, Cloete, Reddy, & Lombard, 2011).

### Methodology

This study relied on qualitative research drawing on in-depth interviews. Qualitative research relies on the purposeful sampling of few individuals to allow for an in-depth understanding of a phenomenon (Palinkas et al., 2015). Small samples have implications connected to the generalisability of findings but in qualitative studies, the most important goal is often to understand behaviour and why people hold certain points of view.

Initial participant recruitment was done using purposive sampling through LGBTIQ (lesbian, gay, bisexual, transgender, intersex, queer) organisations at UKZN and thereafter snowball sampling was employed. The respondents in the initial recruitment sample may have influenced the type of participants selected since the contact was initially made through existing organisations. As participation in this study was voluntary, there was numerous challenges in the recruitment process as many potential participants withdrew from the study citing that the topic made them uncomfortable. Many of these participants who withdrew from the study did not explain themselves although it is possible that as HIV is a sensitive topic and they were not comfortable with discussing their personal life. During this recruitment period, the researcher was introduced to over 30 potential participants but only 15 agreed to participate in the study. Although the study did not intend to recruit participants of any particular population group, all the participants in this study were black Africans. This is due to the demographic composition of the university with black students constituting the majority at 71% followed by Indians at 22% while other racial minorities constitute the rest (UKZN, 2017). Regardless, according to Mutinta et al. as cited in Ngidi, Moyo, Zulu, Adam, and Krishna (2016), black African students are more responsive to HIV studies while HIV in universities remains associated with race and ‘othering’ which makes the recruitment of racial minorities difficult.

The sample in this study consisted of 15 UKZN students between the ages of 18 and 30 who self-identified as MSM. Based on the definition of MSM for this study, the participants were of varying sexual orientations. Most stated that they were currently or had previously been in committed relationships except one man who described his relationship history as complicated. Their level of education ranged from undergraduate to postgraduate. It is worth noting that their ages of did not correspond with their level of study. Some of the students have also been in the university environment for longer but, due to various circumstances they remained in lower levels of study. As a possible limitation, the participants’ level of education and the formal procedures of the interview process may have created an improved sense of the importance of public health among the participants. Table 1.

**Table 1.** Demographic profile of participants.

Characteristics	Categories	N	%
Age	<24	6	40.0
	24+	9	60.0
Sexual orientation	Bisexual	4	26.7
	Gay	10	66.7
	Heterosexual	1	6.7
Relationship status	In a relationship	12	80.0
	Not in a relationship	3	20.0
Education	Undergraduate	8	53.3
	Postgraduate	7	46.7
	** **	**15**	**100**.**0**

This study was conducted as part of a larger study on HIV-risky behaviours among student MSM. In-depth interviews were used to investigate the students’ risky sexual behaviours and included information on perceptions of CHCT. The issue of CHCT emerged as a major theme throughout the interviews although the interview guide used in this study also investigated other domains. These other domains included HIV risk perception, willingness to protect using various methods, attitudes towards risky behaviours such as inconsistent condom use, forced sex and alcohol consumption. The in-depth interviews, which lasted on average one hour, were conducted in English, digitally recorded and transcribed.

### Data analysis

The interviews were analysed using thematic analysis. The first step in data analysis is the transcription and expansion of field notes and audio tapes from the in-depth interviews to increase accuracy (Stuckey, 2014). Data from each of the 15 interviews were analysed separately. This was to ensure an easy comparison of responses under each theme. As a foundational method for qualitative analysis, the main advantage of thematic analysis in this study was flexibility in terms of sorting data into broad themes (Braun & Clarke, 2006). The themes were not pre-determined but rather developed from the codes that were generated during the study. As there was no code list prior to data collection, initial codes were generated in the course of the study. Similar codes were grouped into potential themes of which one of the major themes is discussed in this current study i.e. perceptions on couples’ HIV testing.

### Ethical considerations

Ethical clearance for this study was granted by the University of KwaZulu-Natal’s Humanities and Social Science Research Ethics Committee (Protocol Reference Number: HSS/0205/017M). The ethical procedures adhered to include ensuring the availability of an informed consent form in a language that the participants understood to protect the autonomy of all participants. All participants in this study were assured of confidentiality throughout the research process. They were also required to willingly sign the informed consent form under a pseudonym to protect their identity. The recruitment process ensured dignity and respect. As such, the researcher did not assume potential participants’ sexual behaviour/orientation/gender identity based on observation without the individuals confirming it themselves. Participation in the study was entirely voluntary. The study recognised that some men who have sex with men are possibly reluctant to identify themselves or be identified as MSM due to fear of homophobia, stigmatisation and discrimination and other personal reasons. Individuals who were interested in taking part in the study took the initiative to get in touch with the researcher to express their interest. No remuneration was offered for participation in the study. Although this study was not concerned about sexual orientation, all participants were required to confirm that they had been sexually active with men at some point in their life.

## Findings

### Attitudes towards CHCT

While acknowledging the importance of knowing the HIV status of their sexual partners, only four out of the 15 participants had undergone couples HIV testing with their current or most recent primary partners. The participants were also more likely to have casual partners of unknown HIV status. The majority stated that they could not be sure of their casual and/or primary partners’ HIV status as they did not test together as couples. They expressed fear concerning this lack of certainty about their partners’ HIV status as it was normally verbally communicated or confirmed by the provision of evidence such as a letter from the testing centre.

*With my current partner I haven’t discussed the matter with him but with my previous partner we discussed the issue and tested separately because we are young and we showed each other the results via social media (Njabulo, 23 years)*

*With my partners I have never tested together with them but it is not good because we communicate HIV status verbally so you can never be sure if they are telling the truth or lying to you (Kabelo, 25 years)*

In the interviews, it was clear that MSM appreciate the value of CHCT and hold favourable attitudes to it. They believe that couple testing is important in strengthening the relationship. They identified communication as a key component of a relationship that makes couple testing easier.

*Best thing is to communicate with your partner. When you bring up the topic look at them as a way of assuring them. Then always go and get tested together with them (Kwena, 28 years)*

*Whenever I am in a relationship, I politely say to my partner that ‘okay, since we just met, I would like us to go and get tested and know our status’. The first question is do you know your status? Then I suggest we test together. But before we test, I reassure my partner that the outcome of the test won’t change anything in the relationship and make them see that you understand what HIV is before you go there (Thando B, 24 years)*

*Some of my partners trust me when we test together because if their result comes positive, I assure them that I will stay with them (Panda, 29 years)*

### Reasons for not testing as couples

#### Type of relationship

Decision to undergo couple testing depended on the type of relationship. All the participants displayed a lack of interest in knowing the HIV status of their casual partners. As the majority reserved unprotected intercourse for their primary partners, the purpose of testing for HIV was to establish the need for protective measures in the relationship as it develops. Resultantly, they avoided bringing up HIV testing with their casual partners out of fear that it would be interpreted as a desire to advance the relationship beyond the short-term. They also stated that they did not enquire about the HIV status of their female partners because many of them did not have long-term plans to settle with a woman. Furthermore, they felt protected from HIV by the use of condoms as contraception. The attitudes explained above also partly stem from their belief that condoms provided them with sufficient protection and they, therefore, felt there was no need to worry about the HIV status of short-time sexual partners.

*When I cheat on my [primary] partner I do not bring up the issue of HIV testing [with the partners I cheat with], I just stick to condoms (Handsome, 21 years)*

*Sometimes I am like why bring up testing. He or she will think I want to get married now (Panda, 29 years)*

*If it is a girl I don’t care about testing. I just stick to condoms to avoid getting her pregnant (Mtho, 23)*

#### Perceived and experienced homophobia at public testing facilities

The most accessible health facilities were government-owned public clinics which is why the lack of availability of LGBTIQ-friendly services at these testing centres was a major barrier to CHCT. Unfriendly services included judgmental staff and lack of LGBTIQ-friendly programmes such as regular seminars, particularly on the university campus. The majority of participants stated that despite their willingness to test together with their partners they did not have as much freedom as heterosexual couples. Once they had decided to start engaging in unprotected intercourse, they often resorted to testing separately to avoid being noticed as a couple. Among those who had previously tested with their partners, they did so at LGBTIQ seminars held on campus but due to the low frequencies of such meetings, they could not regularly test with their partners.

*With the current one I didn’t [go for a HIV test] … My partner only sent me a screen grab showing that he went to test and he was HIV negative. So now I feel like it’s important to go with our partners which is not an easy thing because we don’t have so many clinics that support the LGBTIQ community. Unless we are at the seminar where it is only gays and lesbians. It easy to test there. But I am afraid of testing at the clinic because I am afraid of the things they [clinic staff] would say. They should not have [separate] facilities dedicated to LGBTIQ, they should not discriminate against us but the nurses need to be taught about us. You have a STI and they ask where you got it from? And they remind you that it’s because you are sleeping with men that is why you have an STI. Whenever I go to the clinic, I lie that I sleep with women because I am uncomfortable telling them that I sleep with men (Leezy, 23 years)*

*If you tell them the truth, then they [nurses] act like your mother. I never tell them I sleep with men (Sinazo, 19)*

*It [couples testing] is easy if your boyfriend is also at university. Here in Durban, I am only comfortable at the university clinic. They treat us with respect. Outside the university, the nurses are rude if they find out who you sleep with. (Kritik, 27)*

#### Interpersonal relationship factors

Couples testing was also hindered by relationship factors which mainly had to do with trust between partners. CHCT was generally compounded by a general fear of discussing HIV status with sexual partners. The participants stated that bringing up the topic of HIV in a relationship was never easy, particularly in long-term relationships where trust is the basis of the relationship between primary partners. Based on different experiences outlined by the participants, some primary partners felt that talking about testing was a direct admission of infidelity.

*I have been afraid to bring up the topic because you do not know what the other person is thinking when you start talking about it (Kabelo, 25 years)*

*I bring it [couples testing] up, but someone will say so you do not trust me but I trust you. So, if you have a weak spot for that person you avoid talking about it (Kaybee, 21 years)*

*It is not easy to talk about doing HIV tests because some of them are scared of testing positive or they think you are accusing them of sleeping around (Panda, 29 years)*

These interpersonal relationship factors were a major hindrance for CHCT among the 14 participants who admitted to presently or previously have been in serious relationships. These participants cited being in the medical field or being active in HIV-related campaigns at the university as being one of the reasons that have helped them to test together with their partners without causing misunderstanding between them.

*People know my status because I am very involved in the HIV sector. My current partner is also in the same field (Lekko Motion, 26 years)*

*I have tested with a partner before but that time I was young and serious about going to these LGBT events a lot. Sometimes they test us there (Panda, 29 years)*

Another reason for not testing together with their primary partners was the fear of the possibility of a positive result. More than half of the participants stated that they were knowledgeable about their natural risk. The known high HIV prevalence among MSM caused them to anticipate an HIV-positive result with each test. This demotivated them from testing with some of their partners out of fear of having to handle a fragile situation in the event one tested positive. Many opted to test separately but, they remained uncertain whether their partners would be honest in this regard.

*I never talk about HIV at all with the people [casual or primary] I sleep with because I do not feel comfortable discussing it. I only talk about it with my friends (The queen, 25 years)*

*Sometimes I prefer to test separately because I feel that it is the best way to handle the situation in case one is positive and one is negative so the best way is each person for himself (Mtho, 23 years)*

### Alternative strategies to ascertain partners’ HIV status

All participants in this study alluded to the importance of knowing the HIV status of one’s sexual partners to minimise the risk of HIV transmission. In the absence of opportunities to access and undergo CHCT at health care facilities due to the above-mentioned barriers, the participants employed alternative strategies. They believed that these strategies minimised their risk in instances where they could not access CHCT to confirm their partners’ status.

One of the strategies employed was home-based HIV testing which is now available in some leading retailers at affordable prices. Despite lacking the component of counselling, home-based testing was seen as a viable private option. According to those who had used this method before, some of their partners were more open to it than CHCT at public facilities. To overcome the challenges of testing without counselling they stated that it was important to develop open lines of communication with their partners. This was done by bringing up the issue of testing in a sensitive manner as it would assure them that the outcome of the test cannot alter their relationship. In fact, the participants who opted for home testing showed a willingness to be with HIV-positive partners.

*I will not straight up ask you ‘are you clean?’ The way we talk when referring to HIV status we say; ‘Are you clean?’. With some partners it took time and with others it didn’t. Most of the guys I have dated are aware of HIV and STIs so we agree on testing. We buy a kit and test. One of the guys came out positive and I referred him for help but he ended up breaking up with me even though I was willing to continue dating him (Thando B, 24 years)*

*I reassure my partner that the outcome of the test won’t change anything in the relationship and make them see that you understand what HIV is before we test. Make them understand that either of you being HIV positive won’t change how you will go on knowing that one of you has it (Star mor, 24 years)*

Others expressed reservations about home-based rapid tests as they believed that if a person is on antiretroviral treatment and is virally suppressed the test result would report a false negative result.

*The virus can hide and it may not show with a rapid test. I have met a straight person who played girls and wasn’t afraid to test for HIV because he knew it did not show so his result will be negative (Sinazo, 19 year)*

Participants also used other unconventional strategies to use a potential partner’s sexual history to their advantage. They believed that this information could help them to determine if that particular partner put them at increased risk of HIV. These strategies included limiting sexual relationships to individuals already known to them. They mentioned using social media to investigate their potential partner’s relationship history and lifestyle. According to the participants, if a potential sexual partner is very active on different social media platforms and has listed many ex-partners it suggested that he or she is putting them at a higher risk for HIV. If they perceived their partner as increasing their risk of HIV infection they either avoided further contact or religiously used condoms with those particular individuals.

*Date within your circles as in people you know their sex life already. Or get to know the person before anything happens whether it’s a drunk encounter even if he doesn’t tell you his sexual history. You need to observe because sometimes it is obvious, it is clear if he has had too much experience (Mtho, 23 years)*

*Use social media. You can get to know the person before you get to know them. For example, some guys post pictures of their partners and if you find that he has a lot of friends then you know that he has had a lot of sexual partners. That is a safer way of finding out his history. You will know before he confirms or tells you anything. Tthose are the ways I use mostly to protect and prevent myself before I have sex with someone new (Kritik, 27)*

Another strategy employed was the choice to have sexual intercourse with casual partners who belong to specific high-income socio-economic strata that they informally associate with low HIV risk. For example, MSM who are university educated, dress presentably and have a good command of English were supposedly less likely to be HIV positive because it is assumed that they know how to protect their health.

*When you converse in a club or at a party, what you actually do is measure their well-being and you go home with the one whose well-being is okay with you. Sometimes you don’t if his well-being is not good … It is in the way he presents himself, for example, his job, accent, the way he talks or dresses (Njabulo, 23)*

*To be safe [from HIV infection] I will start by getting to know them. Where they are going in life, their aspirations [educational and career]. Positive, inspirational people are the best [safest] (Lekko Motion, 26)*

Participants are aware of the importance of sexual partners being certain of each other’s HIV status to avoid transmission. The findings also show that they appreciate the value of CHCT with their male partners despite being unable freely do so with all their partners.

## Discussion

This study approached CHCT in the context of MSM in universities. This study focuses on a particular group i.e. university students that are less likely to see the need for such services due to their probable relationship dynamics. University students and in particular, MSM students are neglected in CHCT studies possibly due to the low likelihood of them being in long-term cohabiting relationships. The bulk of CHCT literature in sub-Saharan Africans emphasised on married and cohabiting heterosexual couples while general studies on HIV testing and counselling among university students are focused on voluntary individual testing (Desgrées-du-Loû & Orne-Gliemann, 2008; Gedefaw, 2016; Painter, 2001; Peltzer, Mpofu, Baguma, & Lawal, 2002). CHCT of MSM with their regular short-term partners may possibly reduce their risk of exposure. The few existing studies on CHCT among MSM in South Africa have reported high levels of acceptability yet, in contrast, the MSM students in this study did not always test with their partners despite appreciating the value of CHCT (Stephenson et al., 2012; Stephenson et al., 2013).

For many studies, the university environment offers freedom from parental control and for sexual minority students, it is an opportunity to explore their sexuality and discover themselves (Alessi, Sapiro, Kahn, & Craig, 2017). This implies a high rate of casual sex in university environments as many students are at a point in their lives where they are not ready to settle with one partner for life yet they are ready to have intimate relationships (Garcia, Gesselman, Massey, Seibold-Simpson, & Merriwether, 2018). The findings of this study also suggest that MSM students have had several short-term partners. Consistent with past research, this study also found among MSM students CHCT was hindered by its association with intentions to advance the relationship beyond its casual status (Stephenson et al., 2011).

Unlike previously assumed, casual sex is not always meaningless because it is at times as intimate as a primary relationship, especially among university students (Garcia et al., 2018). This suggests a high possibility of MSM students to enter short-term relationships repeatedly and regularly with the same casual partners over long periods. As sexual partners meet regularly, they slowly become less consistent in using condoms. This means that regular short-term and/or casual sex partners can also engage in unsafe sexual practices (Hill, Bavinton, & Armstrong, 2018). It becomes important for prevention programmes to promote couples testing in universities regardless of the type and number of sexual relationships the student MSM have with their partners.

In most of their relationships, the study participants stated that they tested separately and confirmed HIV status verbally upon provision of proof. In their primary and/or committed relationships, the participants cited perceived and experienced homophobia resulting from the limited availability of LGBTIQ-friendly facilities and services. The unfriendly nature of HIV testing centres was attributed to the negative attitudes of health care staff towards same-sex sexual partners. In some cases, the participants were not deterred by lived experiences but rather by their own fears based on the experiences of others. Socio-ecological barriers such as provider mistreatment and confidentiality breaches are a proven barrier to CHCT among MSM (Logie et al., 2017). This finding is consistent with past research which showed that externalised and internalised homophobia are among the major barriers to regular HIV testing (Arnold, Rebchook, & Kegeles, 2014; Wei et al., 2016). In a heteronormative sub-Saharan African context, homophobia (experienced and perceived) is likely to play a major role in determining whether MSM test with their partners. Homophobia has negative implications for HIV prevention as it deters MSM from accessing key health services which are available at university campuses. There is a need to engage MSM students and their partners in couple-based HIV testing programmes without exacerbating the experiences of stigma and discrimination. Integrating efforts against homophobia into existing HIV campaigns will encourage MSM couples to come out and test with their sexual partners.

Another major reason for the lack of CHCT were interpersonal relationship factors ranging from trust, communication and fear of HIV-positive results. In this study, the participants were weary that discord between them and their primary partners would arise if they suggested CHCT. The findings reveal that it is not expected for primary partners who have reached a certain level of trust to seek HIV testing. Suggesting going for an HIV test was seen as an indication of an absence of trust, yet studies have shown that many MSM are infected by their primary partners (Mitchell & Horvath, 2013). As such, this study suggests that there is a need to engage MSM in universities and their primary partners on the importance of regular CHCT.

Some MSM did not test with their partners out of fear of receiving a positive result for either of them in the presence of the other. This contrasts with some past research which suggests that some MSM are willing to use couples testing as a means to disclose sero-status as it provides a forum for the discussion of results among couples (Stephenson et al., 2011). Being university students, the participants have very high levels of HIV awareness and knowledge which is likely to heighten their awareness of their risk of HIV infection. Despite the participants appreciating the value of CHCT and past research suggesting high levels of acceptability of CHCT (Stephenson et al., 2014), they were not willing to deal with the simultaneous discovery of sero-discordance.

The findings also report on alternative strategies employed by MSMs to purportedly know their casual and primary partners’ status. These strategies included a preference for home-based testing kits, reducing HIV risk by engaging with known partners, using social media to investigate a partner’s sexual history and selecting casual partners who appear to belong to high-income socio-economic strata. These alternative strategies can also potentially further heighten their HIV risk. As mentioned by some of the participants, they were uncertain of rapid home-testing kit results because HIV-positive partners are more likely to have a false HIV-negative test result if they are virally suppressed (Merchant, Wright, Kabat, & Yogev, 2014).

Another implication of this finding is that MSM who use store-bought HIV testing kits miss out on a very key opportunity to receive pre and post-test counselling from trained professionals. Counselling has been shown to promote behaviour change and reduce transmission among couples (Peltzer, Nzewi, & Mohan, 2004). Implications for selecting sexual partners from the same social network are that they can potentially create risky sexual networks. This is possible through the increased likelihood of partner exchange which may result in the rapid spread of HIV in that social circle. Evidence suggests that among African MSM, members of the same social network often share similar norms, attitudes and HIV risk behaviour; thus the vulnerability of an individual increases when they enter a high-risk sexual network (Amirkhanian, 2014). In the context of this study, institutions of higher learning should implement network interventions among MSM students. These intervention networks can be designed in such a way that they seek to understand MSM communities in universities to identify and recruit MSM students who are unlikely to be reached by the conventional HIV-related programmes already existing on university campuses (Amirkhanian, 2014). This will potentially reduce risky behaviour and promote the uptake of CHCT in a population group that has low levels of couples testing.

In this study, participants believed that people who are very active on social media platforms are more likely to be HIV positive. In the past, MSM have been shown to use internet platforms to employ risk-reduction strategies such as sero-sorting where they partner with people of the same HIV status (Siegler, Sullivan, Khosropour, & Rosenberg, 2013). In this study, the method used by the participants to investigate their partners’ HIV status is a misguided assumption that people who are not very active on social media will not expose them to HIV. The study participants associated low socio-economic status with high HIV risk. This notion is supported by past research which suggests that socio-economically disconnected MSM have higher rates of HIV than their peers (Gayles, Kuhns, Kwon, Mustanski, & Garofalo, 2016). Regardless, there is no evidence in the literature to suggest that there exists a strong link between socio-economic status and HIV status. Informal assumptions such as these, expose MSM in universities to high HIV risk as they see no need to test with partners who fit a certain criterion.

This study draws attention to CHCT testing among MSM in university- a group that is less likely to have long-term partners. The findings presented are not exhaustive in exploring the contextual relationship backgrounds of the student MSM who were interviewed. For example, the study does not enquire about the specific HIV testing practices of participants in their relationships with their partners (past, present and concurrent). This is because most of the participants were not willing to be open about the number and type of sexual partners in their lives. The study is also limited by its sample size of 15 black African students which affects its generalisability to other sub-populations of MSM students. This study mostly discusses the findings in the context of general MSM populations because very little research has been done on MSM in the university context.

## Conclusion

The study reveals that MSM in university appreciate the value of CHCT with their primary partners whilst they deem it unnecessary with casual partners. They associated CHCT with the desire to make the relationship long term. At the same time, within their primary relationships, they were not always able to test with their partners citing homophobia at testing facilities and interpersonal relationship factors. In the absence of CHCT, the participants turned to home-based testing using rapid test kits sold in some leading retailers. MSM also found other ways to reduce their risk such as having sexual encounters with people from familiar social networks, investigating their partners’ sexual history via social media and filtering out potential casual partners from lower socio-economic backgrounds. These findings highlight the importance of CHCT among MSM in universities. Educating MSM in universities on the importance of CHCT may result in behaviour change with long-term and short-term partners of unconfirmed HIV status. Based on the findings of this study, we can conclude that future research should uncover different ways of promoting CHCT among MSM in primary or casual relationships in the general and university populations.
